# Repetitive DNA Sequences in the Human Y Chromosome and Male Infertility

**DOI:** 10.3389/fcell.2022.831338

**Published:** 2022-07-13

**Authors:** Yong Xu, Qianqian Pang

**Affiliations:** ^1^ Department of Emergency Surgery, Jining NO 1 People’s Hospital, Jining, China; ^2^ Institute of Forensic Medicine and Laboratory Medicine, Jining Medical University, Jining, China

**Keywords:** repetitive DNA sequences, Y chromosome, copy number variation (CNV), azoospermia factor region (AZF), testis-specific protein Y encoding, male infertility

## Abstract

The male-specific Y chromosome, which is well known for its diverse and complex repetitive sequences, has different sizes, genome structures, contents and evolutionary trajectories from other chromosomes and is of great significance for testis development and function. The large number of repetitive sequences and palindrome structure of the Y chromosome play an important role in maintaining the stability of male sex determining genes, although they can also cause non-allelic homologous recombination within the chromosome. Deletion of certain Y chromosome sequences will lead to spermatogenesis disorders and male infertility. And Y chromosome genes are also involved in the occurrence of reproductive system cancers and can increase the susceptibility of other tumors. In addition, the Y chromosome has very special value in the personal identification and parentage testing of male-related cases in forensic medicine because of its unique paternal genetic characteristics. In view of the extremely high frequency and complexity of gene rearrangements and the limitations of sequencing technology, the analysis of Y chromosome sequences and the study of Y-gene function still have many unsolved problems. This article will introduce the structure and repetitive sequence of the Y chromosome, summarize the correlation between Y chromosome various sequence deletions and male infertility for understanding the repetitive sequence of Y chromosome more systematically, in order to provide research motivation for further explore of the molecules mechanism of Y-deletion and male infertility and theoretical foundations for the transformation of basic research into applications in clinical medicine and forensic medicine.

## 1 Introduction

**FIGURE 1:**
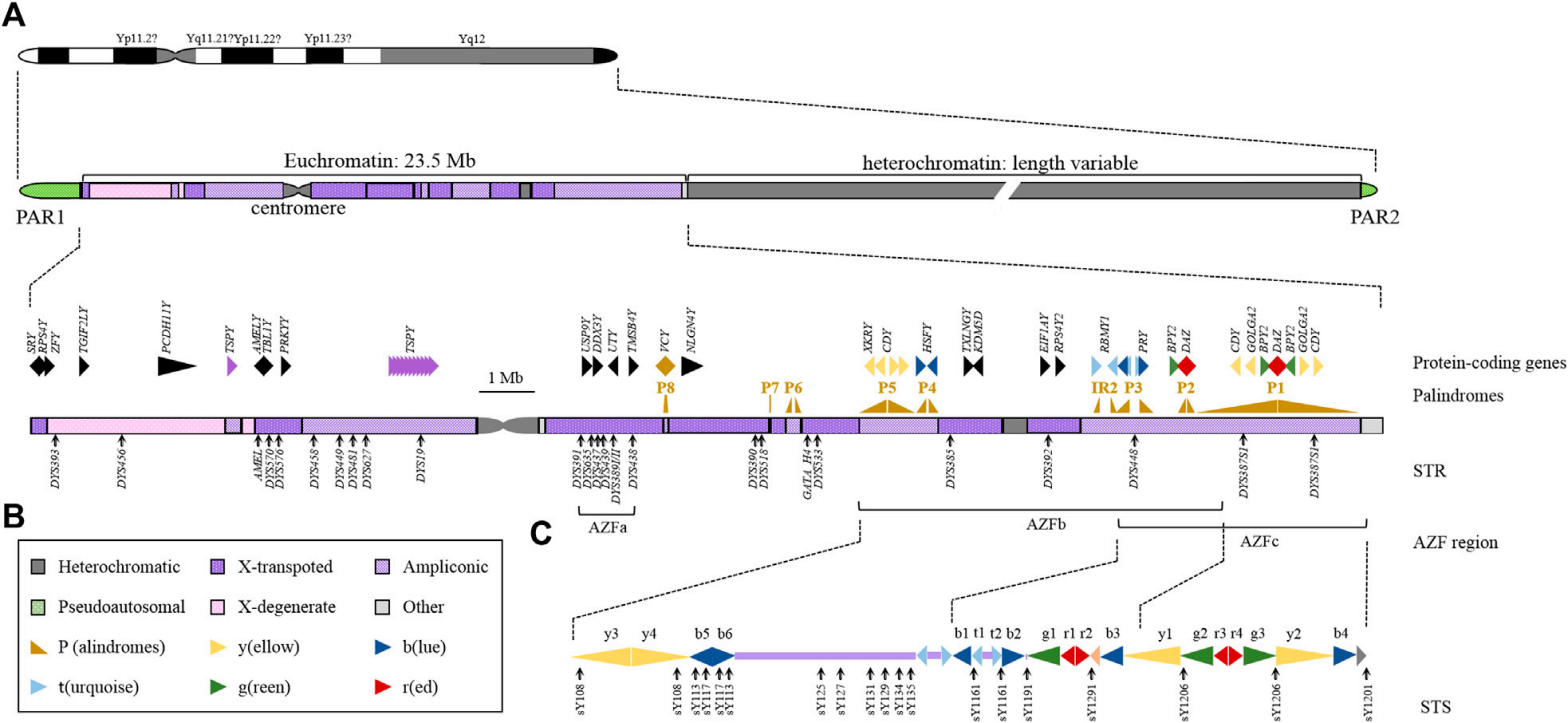
Structural and Genetic Markers of the Y Chromosome. **(A)** Ideogram of the human Y chromosome. Different structures of chromosomes are represented by different colours. **(B)** Expanded view of the human MSY euchromatin. The arrowheads indicate the transcriptional direction of the palindromes and genes. The black solid triangles show the single-copy genes, and the colours represent the multicopy genes. The gene name is marked above the symbol. The black arrows below the chromosomes indicate the locations of STRs used in forensic science and their names. **(C)** Schematic organization of the AZFb and AZFc region. Repeating units are represented by solid triangles of different colours, and arrowheads point to the transcriptional direction. The black arrows below the chromosomes indicate the locations of STSs and their names.

There are a large number of palindrome and repetitive sequences in the Y chromosome that are unique to men. Due to the limitations of sequencing technology, Y-DNA repetition is still one of the most mysterious parts of the eukaryotic genome. The human Y chromosome is significantly different from other chromosomes in terms of size, genome structure, content, inheritance mode, and evolutionary path (Navarro-Costa, 2012). From a genetic point of view, the Y chromosome is haploid, except for the two pseudoautosomal regions (PAR1 and PAR2), and most of the sequences that exclude recombination with the X chromosome occur in the male-specific Y region (MSY), which accounts for approximately 95% of the length of the entire Y chromosome (Deschepper, 2017). PAR corresponds to the X-Y homologous block, contains 27 genes that encode products related to different biological functions and has a corresponding variable expression pattern (Helena Mangs and Morris, 2007). PAR1 is involved in meiotic pairing, which is a necessary process for successful male meiosis (Kauppi et al., 2011; Krausz and Casamonti, 2017). The MSY region rarely recombines with the X chromosome, and all Y- short tandem repeats (STRs) are inherited in the linkage, which is the molecular basis for the Y chromosome to maintain paternal inheritance in the form of a haplotype (Figure 1). This genetic feature has very important practical significance for the identification of male-related cases in forensic medicine, and the special structure and function of the Y chromosome make it unique in the field of forensic medicine research.

However, the MSY sequence is not completely isolated from the X chromosome. The existence of a large number of regions that are highly homologous to the X chromosome or autosomes means that recombination can take place in these regions. MSY can be further divided into euchromatin regions and heterochromatin regions. The heterochromatin region is composed of highly repetitive sequences. Due to technical limitations, accurate sequencing has not yet been possible. The euchromatin region contains three types of sequences: X-transposed, X-degenerate and ampliconic. X-transposed maintains 99% identity with the X chromosome, and this sequence can undergo homologous recombination with the X chromosome (Krausz and Casamonti, 2017). The X degenerate region mainly contains single copy genes or pseudogene homologues of X-linked genes (Skaletsky et al., 2003). The amplicon region contains multiple copies of genes specifically expressed in the testis, but Y is still the chromosome with the lowest gene density (Hallast et al., 2021). Due to sequence homology, there will also be a certain degree of exchange between the Y chromosome and other chromosomes. In addition, the changes in the satellite array inside the Y chromosome have been shown to affect the size of the entire Y chromosome in the population. The frequency of satellite mutations may fluctuate greatly between individuals, and Y satDNA would represent a valuable tool for studying human mutations.

## 2 Repetitive Sequences of the Y Chromosome

The uniqueness of the Y chromosome is that its structure mostly consists of highly repetitive sequences, primarily repeats and palindromic sequences with forward or reverse repetitions. In addition to the traditional centromere and telomere regions, the Y chromosome also contains a large number of repetitive sequences on the long arm. Eight large-scale palindrome structures, namely, the P1-P8 palindrome (Figure 1), are mainly observed. Moreover, the large palindrome structure also contains small palindrome sequences. Although the palindrome structure of these eight regions is not a perfect palindrome sequence, the consistency of their symmetrical sequences on both sides reaches 99.9% (Dweck and Maitra, 2021). In addition, the amplicon region also contains multiple larger inverted repeats with low sequence identity. The most studied repetitive sequences mainly include the azoospermia factor region (AZF) region, testis-specific protein Y encoding (TSPY) cluster and other multicopy gene regions. Repetitive sequences can increase the expression dose of genes and provide an appropriate nuclear chromatin environment for germ cells to prevent gene loss during multigenerational transmission. On the other hand, it may also make the Y chromosome prone to extensive changes in the number of gene copies in these repetitive sequences. The presence of these repetitive sequences allows a mechanism called nonallelic homologous recombination (NAHR), which may affect the deletion/repetition of gene dose caused by changes in the number of repetitive sequences on the Y chromosome, that is, copy number variation (CNV) (Cioppi et al., 2021). Among all human chromosomes, most CNVs are located on the Y chromosome (Freeman et al., 2006). Although CNV is not unique to the Y chromosome (Singh et al., 2019), it has an impact on spermatogenesis and is a well-known cause of male infertility.

It should be noted that the sequence, structure, and copy number of multiple-copy genes are different in different Y chromosomes, and there will also be significant differences in people with different genetic backgrounds. Because of the presence of repeats, deciphering the function of the Y chromosome in mammals has been very slow. Previous studies have focused more on Y chromosome microdeletion as a risk factor for spermatogenesis failure (SF). In addition, the long arm of the Y chromosome contains multiple large palindromic structures, especially the AZF region, which contains a large number of multiple copies of genes. At present, research on AZF-related duplication or complex CNV (deletion + duplication) is booming. It is currently believed that Y chromosome CNV is mostly related to spermatogenesis and may also be involved in the occurrence and development of cancer. Yq deletion has been confirmed to have a clear causal relationship with severely impaired spermatogenesis. In the CNV analysis of MSY, Yp duplications containing amelogenin Y-linked (AMELY) and TBL1Y genes, partial deletions of TSPY clusters, RBMY gene numbers and duplication of two fragments in the AZFa region have been reported (Krausz and Casamonti, 2017). Infertile men with azoospermia or severe oligospermia have a higher incidence of chromosome abnormalities and Y chromosome microdeletions in China. Research on spermatogenesis and Y chromosome deletion has great clinical significance. At present, the screening of AZF deletion has become a part of routine diagnostic examinations for men with severe oligospermia/azoospermia (Krausz et al., 2014). With more thorough research on the function of repetitive sequences, increasingly detailed data have been gradually obtained, thus providing a scientific basis for promoting the genetic screening of infertile men before assisted reproductive technology (ART) (Fu et al., 2012).

### 2.1 AZF Region

AZF microdeletion is the most common chromosome structural abnormality and the main cause of male infertility associated with Y chromosome CNV. Although AZF loss has become an important factor in severe oligospermia/azoospermia, there are still some clinical issues that need further research. Due to the huge impact on reproductive ability, AZF microdeletion usually occurs *de novo* by NAHR between sister chromatids in the father’s testis during sperm meiosis (He et al., 2020). The AZF region has been shown to contain important spermatogenesis-related genes and gene families (Krausz and Casamonti, 2017). The multicopy nature of multiple genes increases the difficulty of identifying genes that cause related phenotypes (Cioppi et al., 2021).

#### 2.1.1 Structure

According to the initial identification, the AZF region contains three members: AZFa, AZFb, and AZFc. In terms of positioning, AZFa is closer to the centromere on the long arm of the Y chromosome while AZFb and AZFc are on the long arm closer to the telomere and have more complex repetitive regions. Some researchers refer to the shared region of AZFb and AZFc as AZFbc.

The AZFa region is 792 kb long and encodes only a single copy of the gene. Currently, four genes are located in the AZFa region, while homologous genes are observed on the X chromosome to avoid inactivation. Among them, USP9Y and DDX3Y are ubiquitously expressed single-copy genes. The single deletion of USP9Y is related to a wide range of testicular phenotypes that range from azoospermia with insufficient spermatogenesis to normal spermatism (Tyler-Smith and Krausz, 2009). Rather than being a functional gene, USP9Y is more likely to be a fine-tuner to improve efficiency. Although human individuals carrying mutations or deletions of the DDX3Y gene have not been reported, knockout of the DDX3Y gene can also cause reproductive disorders, namely, sertoli cell only syndrome (SCOS), which is characterized by no germ cells in the testis, a low testis volume, and high FSH (Cioppi et al., 2021). UTY encodes a male-specific histone demethylase that can catalyse the demethylation of H3K27me3 in histone H3. UTY may also act as a chaperone to participate in protein–protein interactions, induce transplant rejection of male stem cell transplants, and participate in a transcriptional regulatory network that is essential for prostate differentiation (Walport et al., 2014; Dutta et al., 2016; Nailwal and Chauhan, 2017b). However, the role of UTY in the testis is unclear. TMSB4Y encodes a new human leukocyte antigen, namely, a minor histocompatibility antigen, and is a key activator of natural killer cell cytotoxicity; however, its involvement in testicular function is unclear (Torikai et al., 2004; Kumar et al., 2018).

**FIGURE 2:**
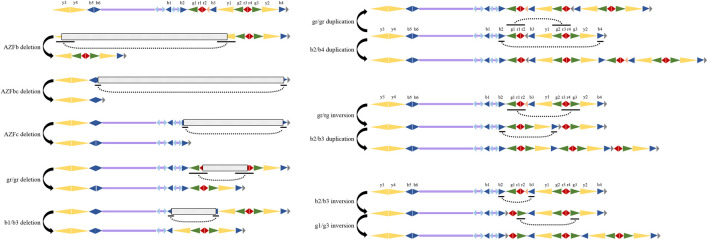
CNV in AZFb and AZFc of the Y Chromosome. Repeating units are represented by arrows of different colours, and the size and transcriptional direction of the amplicons are represented by the length and direction of the arrows. The left column is the deletion type, and the right column is the inversion and duplication. The dashed curve indicates the position of the recombination. The shaded block describes the location of the deletion. Map information is adapted from published data.

AZFb is 6.2 Mb and contains palindromes P2 to P5 and the proximal part of P1 (Figure 1). The region has a complex genomic structure, including 4 single-copy genes and 16 amplicons. These amplicons are all multicopy sequence units and can be organized into six sequence families (identified by different colours in Figure 1 and Figure 2) according to the high-order structure of a symmetrical array of continuous repeating units of palindrome, and the level of sequence homology within the family is extremely high (> 99%). Among the 14 amplicon units, AZFb independently contains 7 (y3, y4, b5, b6, b1, t1, and t2), and the rest are shared with AZFc (approximately 1.5 Mb). The presence of a large number of amplicon domains in AZFb allows for very complex rearrangements. The key region required for spermatocyte maturation extends from the centre of palindrome P5 to the proximal edge of P3 in the RBMY1 cluster and contains 13 coding genes (Kichine et al., 2012). In addition to RBMY, XKRY, HSFY, EIF1AY, RPS4Y2 and PRY are also considered functional genes required for spermatogenesis, and they are also involved in fertility (Vogt, 1998; Ferlin et al., 2007; Lopes et al., 2010; Yamauchi et al., 2014; Yu et al., 2015; Halder et al., 2017). Maybe, it is common knowledge that the AZFb contains several genes of importance, some involved in crucial biological processes such as RBMY, HSFY, EIF1AY, RPS4Y2. However, at present, only the correlation between genes and diseases has been determined, and there is no mouse experiment to verify the expression of genes and confirm the regulatory effect and molecular mechanism. Even overexpression of RBMY in XY(d1) mice cannot rescue the phenotype of abnormal sperm (Trombetta et al., 2017). CNV is not equivalent to gene expression, and CNV research should consider the expression regulation of this gene and downstream target genes more. Therefore, little is currently known about the biological functions and molecular mechanisms of regulation of these genes.

The AZFc region spans 4.5 Mb and encodes 21 candidate genes and 11 transcription unit families, all of which are multicopy genes specifically expressed in the testis (Rozen et al., 2012; Yu et al., 2015). Six different inverted repeat amplicon families composed of palindrome repeat units are arranged in complex repeating patterns to form three large palindromes, which may be formed due to repetitions and tandem inversions in the evolutionary process (Nailwal and Chauhan, 2017a). Among the various transcription units, only four active copies of protein-coding gene families are mapped to the AZFc region, including PRY2, BPY2, DAZ, and CDY1 (Figure 1). The AZFc region contains 4 copies of DAZ, which encodes an RNA binding protein important for spermatogenesis in spermatogonia and is considered to be essential for germ cell development (Kee et al., 2009; Alechine and Corach, 2014). Infertile men with missing copies of DAZ are highly susceptible to azoospermia or severe oligospermia. Ten percent of male spermatogenesis defects have DAZ deletions (Sen et al., 2015). However, there is a lack of evidence for a direct link between DAZ deletion and azoospermia due to the functional homologue (DAZLA) on human chromosome 3. Similar to the DAZ family, CDY, which had four copies, also showed autosomal homologues. In addition to its function as a histone acetyltransferase, little is known about the molecular function of CDY. The BPY2 gene has three copies and is specifically expressed in the testis. Its protein product is believed to be involved in the development of male germ cells by regulating the cytoskeleton in spermatogenesis. GOLGA2LY is arranged in opposite directions in P1 with two copies. Reports have indicated that the gene is only transcribed in the testis and expresses a protein of 108 amino acids (Skaletsky et al., 2003; Jangravi et al., 2013; Alechine and Corach, 2014), although the two copies may have different roles in spermatogenesis (Noordam et al., 2011; Lu et al., 2014). GOLGA2 is a Golgi matrix protein that is essential for the processing and transport of specific proteins by the Golgi (Liu C. et al., 2017; Pang et al., 2021b). Moreover, the deletion of GOLGA2 can cause male sterility by regulating the function of the acrosome (Han et al., 2017). The putative functions of other genes remain unknown, and future studies could provide answers.

#### 2.1.2 Types and Mechanism of CNV

All currently known AZF microdeletions are caused by recombination errors within the chromosome (Krausz and Casamonti, 2017). Because of the nature of the regional duplication and palindrome of AZF region and the fact that it borders the highly repetitive heterochromatin region of Yq12, the AZF region frequently undergoes NAHR and thus is extremely susceptible to the influence of chromosomal rearrangement to produce deletions, duplications and CNVs during meiotic recombination (Freeman et al., 2006). The recombination caused by the HERV15 provirus resulted in the deletion of the AZFa region from the human Y chromosome, and the recombination of P5 and P1 palindromes caused massive AZFb- and AZFc-related deletions (Signore et al., 2019). Most AZFb deletions are caused by NAHR events between identical sequence blocks located in the proximal arm of the P5 palindrome and proximal P1 palindrome. AZFb deletions, also known as “classical” deletions, are caused by the exchange of homologous fragment ends between amplicon y3 in the AZFb region and amplicon y1 in the AZFc region. This AZFb deletion pattern loses the 6.23 Mb sequence containing the shared region of AZFb and AZFc, including the amplicon y4-y1 and at least 32 coding genes, which leads to meiotic arrest (MA) in spermatogenesis (Repping et al., 2002; Ravel et al., 2009). The other is a partial deletion of AZFb with a smaller length of the “classical” AZFb deletion molecule, also known as the “nonclassical” AZFb deletion. It has different proximal and/or distal breakpoints and is therefore associated with variable testicular pathology, including MA, cryptospermia, severe oligospermia oligospermia or oligoasthenoteratozoospermia (OAT) syndrome (Vogt et al., 2021).

The simultaneous deletion of AZFb and AZFc occurs at the two breakpoints between P4/distal P1 (7.0 Mb and 38 gene copies removed) or P5/distal P1 (7.7 Mb and 42 gene copies removed) (Colaco and Modi, 2018).

Because the AZFc region has a large span and contains many repetitive sequences, the type of AZFc deletion is more complicated. The complete deletion of AZFc involves the deletion of a 3.5 Mb region, including amplicons b2 and y2, caused by the recombination of b2 and b4, which contains 12 genes and multiple copy number transcription units (Figure 2). In addition, there are many partial deletions in the AZFc locus, including the most common 1.6 Mb gr/gr deletion caused by the recombination of amplicon sets g1r1r2 and g2r3r4, the b1/b3 deletion type caused by the recombination of amplicon b1 and b3, rare b2/b3 deletions and b1/b2 deletions, RBMY1 gene deletions, etc (Zhou et al., 2019; Hallast et al., 2021). Reports have indicated that three different types of gr/gr deletions are caused by homologous recombination of the amplicons g1/g2, r1/r3, and r2/r4 of the P1 and P2 palindromes in AZFc. However, the removed sequences of these three deletion types are almost the same. By overcoming the limitations of the sequence-tagged site (STS)-PCR technique commonly used to detect AZF deletions, Bunyan et al. used multiplex ligation-dependent probe amplification (MLPA) in 100 subjects to identify four types of deletions that could not be detected by STS-PCR (Bunyan et al., 2012).

Based on a similar mechanism as deletion, recombination between repeated sequences can also cause sequence repetition or inversion and sometimes triple repeats or inverted compound repeat sequences. Later, Saito et al. used the same technique to identify AZF-related deletions and duplications in the Japanese population (Saito et al., 2015). Through a resequencing study of the AZFc region combined with a gene dose analysis of the multicopy genes DAZ, BPY2, and CDY and the Y haplogroup determination, it was found that the Y lineage R1a1-M458 has a greater tendency to carry r2/r3 inversions of approximately 1.6 Mb in Estonian males. This phenomenon reduces the stability of the AZFc region, which in turn causes the severity of oligospermia to increase as the subsequent recurrent microdeletion becomes more complicated (Hallast et al., 2021). Through an next-generation sequencing (NGS) reading depth analysis, Ravasini et al. found two simultaneous NAHR events that contained an interchromatidic 1.6 Mb b1/b3 deletion and an interchromatidic 3.5 Mb b2/b4 duplication (Figure 2), which may have led to DYS448 deletion and DYF387S1 duplication (Ravasini et al., 2021). In recent years, the development of new technologies such as MLPA, NGS, resequencing, aCGH and ddPCR has led to the continuous detection of a large number of new CNV types, but the formation mechanism is not yet clear (Peichel et al., 2020 ; Guevara-Fujita et al., 2021 ). Due to the complex structure of NAHR, the Y chromosome is considered “fragile”. A MLPA analysis of 402 fertile healthy male controls and 423 idiopathic infertile SF patients in the Han population identified a total of 24 types of AZF-linked CNVs. Corresponding to common reported partial deletions of AZFc, duplication of gr/gr, b2/b3, b1/b3, some single genes (such as RBMY1), b2/b3 triplication and three types partial duplication of BPY2 have also been found, and two kinds of duplication will occasionally occur simultaneously. Partial duplication will also occur in the AZFa area. Some complex CNVs of deletion + duplication were also discovered at this time (Zhou et al., 2019; Liu et al., 2020). In addition, the recombination between the repetitive sequences will also invert the sequence of this region and generate some complex CNVs with overlapping inversion and deletion/duplication. The gr/rg inversion caused by the recombination of g1r1r2 and g2r3r4 and the possible subsequent b2/b3 duplication have been reported. It is also possible that b2/b3 inversion will occur first, accompanied by g1/g3 duplication (Lu et al., 2014).

#### 2.1.3 Phenotypic Effects of Spermatogenesis

The study found that the incidence of azoospermia factor (AZF) microdeletion in patients with azoospermia and severe oligospermia was 11.75 and 8.51%, respectively, and the deletion of AZFc was the most common (Fu et al., 2012). The AZFa and AZFb regions are essential for initiating spermatogenesis, while the AZFc region is essential for completing the spermatogenesis process. Generally, AZFa deletion causes SCOS, AZFb deletion causes MA, and AZFc deletion causes hypospermatogenesis with gradual severity, including azoospermia (Vogt, 1998).

The deletion of the entire AZFa area will always lead to SCOS and azoospermia (Kleiman et al., 2012). All patients with AZFa deletions (including AZFa, AZFbc and AZFabc deletions) have azoospermia, and some AZFa deletions are associated with phenotypes ranging from azoospermia to euzoospermia (Wei et al., 2015). This finding indicates that the reduction in genetic content is the key to azoospermia caused by AZFa deletion.

Patients with AZFb region deletion have a mature stagnant testicular phenotype, usually at the spermatocyte stage, and there are no postmeiotic germ cells in most tubules (Navarro-Costa et al., 2010). Depending on the location of the break sites of AZFb partial deletion, they are related to different degrees of testicular lesions, including MA, cryptospermia, severe oligospermia or OAT syndrome, and some patients will produce low numbers of mature sperm (Vogt et al., 2021).

AZFc is the most frequently deleted region of the AZF locus in infertile men (Skaletsky et al., 2003; Colaco and Modi, 2018). Patients with AZFc deficiency usually have lower sperm levels in ejaculate or testicular tissue, but data in the literature indicate that the sex chromosomes of a large proportion of sperm from carriers of AZFc microdeletion are invalid (Jaruzelska et al., 2001).

Partial deletion of the AZFc locus is related to infertility, but the data show that this effect is related to the population (Colaco and Modi, 2018). The association between partial deletion of AZFc and male infertility is not as certain as complete deletion. Studies have shown that men with fertile and normal sperm may also carry partial deletions of AZFc. This is mainly due to the widespread heterogeneity of the missing types of AZFc (Figure 2). The recombination of the repetitions and/or the palindrome sequence within the AZFc region is the most likely reason for this region to be deleted, and it is also the reason why the deletion only changes the copy number of the gene without accompanying the deletion of the gene. Regardless of the deletion type and population, the loss of gene copies in AZFc will increase the individual’s susceptibility to reduced sperm counts. A number of studies have shown that the reduction in the gene dose contained in the AZFc region caused by the gr/gr deletion is correlated to a certain extent with the reduction in sperm count. However, the gr/gr deletion can also be detected in fertile men and men with normal sperm counts (Repping et al., 2003; Stouffs et al., 2011; Bansal et al., 2016). The current data cannot determine the association between gr/gr deletion and male infertility, which may be related to the genetic background (geography and ethnicity) of the population. For example, the Y chromosome haplogroup will cause differences in the susceptibility of gr/gr deletion in male infertility patients (Navarro-Costa et al., 2010). It is also believed that the lack of gene copies of men with gr/gr deletion itself is heterogeneous, such as the copy number of the DAZ and CDY genes. Estimates indicate that only those men who have deleted two copies of DAZ and one CDY will have azoospermia or oligospermia. Keeping any of these genes will hardly affect spermatogenesis and sperm motility (Noordam et al., 2011; Sen et al., 2015; Alimardanian et al., 2016). Moreover, the copy number changes of GOLGA2LY and BPY2 have a certain impact on the fertility of men lacking gr/gr (Noordam et al., 2011; Lu et al., 2014; Sen et al., 2016). Therefore, the copy number changes and gene dosage effects of other genes in the AZFc locus seem to have a synergistic regulatory effect on the fertility of men with gr/gr deletion. Regardless of these special cases, the sperm count and motility of the vast majority of men with gr/gr deficiency are significantly reduced (Stouffs et al., 2011; Rozen et al., 2012; Bansal et al., 2016). The gr/gr deletion may reduce male reproductive ability by hindering spermatogenesis and reducing sperm count or motility. Since b1/b3 deletions are very rare in most people, few research reports have focused on these deletions (Liu et al., 2020). Unlike the gr/gr deletion, this deletion contains part of AZFb and causes the loss of six copies of RBMY1 and two copies of PRY and increases the risk of sperm failure, although its relationship with male infertility is unclear. A Chinese man has been reported to carry b1/b3 deletions, which had a limited effect on spermatogenesis; however, this man is infertile due to a congenital bilateral vas deferens deletion (Kuroda et al., 2018). The b1/b2 and b1/b3 deletions are rare and are currently only detected in male infertility patients, suggesting that the deletions have an impact on spermatogenesis. (Cong et al., 2010 ; Guo et al., 2020). The 8 SCOS patients with total germ cell deletion detected by aCGH all had double CDY1a and CDY1b deletions, with deletions of one or more other genes including DAZ, BPY, CSPG4LYP1, and GOLGA2LY, respectively. Although CDY is critical for spermatogenesis, it is not the only determinant (Peichel et al., 2020). It is worth noting that the current studies have shown that many simple partial deletions of AZFc (gr/gr deletion, b2/b3 deletion and b1/b3 deletion) are not directly related to infertility. CNV in the AZF region is involved in spermatogenesis. The effect is highly dependent on genetic background, such as b2/b3 deletion being completely fixed in Y haplogroup N3 (Hallast et al., 2021). This may be explained by the diversity of gr/gr and b2/b3 deletion subtypes. Complex CNV (b2/b3 deletion + DAZ1/2 duplication) has been proven to increase the genetic risk of SF in the Han population (Zhou et al., 2019). With the development of technology and the deepening of research, CNV typing will become more detailed and systematic and a more definite and clear understanding of the relationship between CNV and spermatogenesis and its regulatory mechanism will be developed.

#### 2.1.4 Medical Applications

Since the loss of genetic content in the AZFa region is a key determinant of azoospermia, the detection of microdeletions in the AZF region is of great significance from a diagnostic point of view. Deletion screening will help clinicians determine the causes of male infertility and develop reasonable strategies of ART for patients (Krausz, 2020; Katagiri and Tamaki, 2021). Since these deletions are 100% passed on to male offspring born through assisted reproduction, testing for the deletions will enable couples to make informed choices regarding the continuation of male infertility in their offspring. In order to avoid such a situation, the patients with this type of microdeletion can be selected without preimplantation genetic diagnosis (PGD) technology—commonly known as “third-generation IVF”—to select embryos without Y chromosomes, so that the inheritance of the microdeletion can be blocked. At the same time, to avoid unnecessary treatment and vertical transmission of genetic defects, before using intracytoplasmic sperm injection (ICSI) for patients with severe spermatogenesis defects, they should carefully evaluate chromosomal abnormalities, perform Y chromosome microdeletion screening and use *in vitro* fertilization (IVF)- embryo transfer genetic counselling before proceeding (Krausz and Riera-Escamilla, 2018; Katagiri and Tamaki, 2021).

AZFa causing SCOS and AZFb causing MA indicate that the diagnosis of complete absence of the AZFa and AZFb region prevents the retrieval of testicular sperm for ICSI. AZFc causes hypospermatogenesis with gradual severity, including azoospermia, and a low sperm level; therefore, AZFc microdeletion has no significant effect on IVF results (Vogt, 1998; Totonchi et al., 2012). Therefore, in these patients, sperm is almost impossible to find in the testicles, even when using sperm collection methods. For such men who want a child, sperm donation or adoption is the only viable option (Cioppi et al., 2021). For carriers of the AZFc microdeletion, sperm can be extracted from the testis through testicular sperm extraction, with a success rate of 50–60% (Flannigan and Schlegel, 2017). In fact, combined with testicular biopsy and ICSI, many men with azoospermia may now have their own children (Tournaye et al., 2017). However, new data on the relationship between Yq deletion and testicular cancer and neuropsychiatric diseases are beginning to emerge. For infertile men with Yq deletion, long-term follow-up data are needed to confirm the actual association (Colaco and Modi, 2018; Moreno-Mendoza et al., 2019; Patel et al., 2019). At present, the clinical detection of Y chromosome microdeletion mainly uses STS-PCR technology. According to the Y chromosome microdeletion detection guidelines jointly published by EAA/EMQN in 2014, the detection site includes 6 basic Y chromosome-specific STS loci (sY84, sY86, sY127, sY134, sY254, sY255, AZFa, b, c each two sites) and autonomously selected extensibility STSs (sY82, sY83, sY1064, sY1065, sY1182, sY88, sY105, sY121, sY1224, sY143, sY1192, sY153, sY160). However, due to the variety and complexity of CNVs in the AZF region, especially AZFc, even the expansion sites is far from sufficient for genotyping (Esteves et al., 2017 ; Yamaguchi et al., 2020 ; Zhang et al., 2021).

### 2.2 TSPY Region

The TSPY gene array is located on the Yp arm. The TSPY gene copy occupies a 20.4 kb sequence in the form of tandem repeats. The copy number of these repetitive sequences is 11–76, and there are large differences between different Y haplogroups (Mathias et al., 1994). No copy number outside this range is found in the current population, which may have a certain impact on individual survival or key developmental processes (Rogers, 2021). Abnormal translocation between the homologous genes PRKX and PRKY can also lead to the loss of TSPY (Nakashima et al., 2013).

A comparison search in GenBank found that TSPY is conserved in Bovidae, and its expression dysregulation is closely related to hybrid male sterility (Zhang et al., 2019). A study in the Czech Republic in 2006 indicated that the copy number ranged from 30 to 60, and the increase in TSPY copy number resulted in a significant increase in infertility. In 2009, the results of a study of Italian men showed that their copy number ranged from 21 to 35, and the lower copy number of TSPY would lead to a significant decrease in sperm count and fertility (Giachini et al., 2009). However, a third study conducted on Dutch men in 2010 found that TSPY copy number had no effect on TSPY copy number and fertility when comparing fertile and infertile men (Nickkholgh et al., 2010). The team conducting the Italian study subsequently proposed that it is important to control the Y haplogroup during these studies due to the differences between the populations. The group expanded their initial study and increased the scale of their study to 212 men with abnormal semen parameters and 168 men with normal semen parameters, and they found that the number of TSPY copies in the infertility group was significantly reduced (Krausz et al., 2011). A study conducted in China in 2013 examined 2,272 Han men and found seven different Y haplogroups. These haplogroups have significantly different average copy numbers of the TSPY gene. Compared with ordinary men with TSPY copy numbers of 21–35, men with fewer than 21 copies or more than 55 copies have significantly lower sperm production and increased opportunities for spermatogenesis (Shen et al., 2013; Flannigan and Schlegel, 2017). To date, the TSPY gene is the most obvious example of CNVs with a significant impact on spermatogenesis. TSPY CNV affects the susceptibility of individuals to SF by regulating the efficiency of spermatogenesis and has a significant quantitative impact on the phenotypic expression of gr/gr deletion. The investigation found that the TSPY copy number distribution was significantly different in non-AZFc-deficient men with different spermatogenic phenotypes. Males with fewer than 21 copies of TSPY or more than 55 copies had lower sperm yield and a higher risk of SF. Similar results were also observed in males with gr/gr deletions. This CNV is the first independent genetic factor that has been clearly observed to affect the spermatogenesis status of carriers with gr/gr deletion (Shen et al., 2013).

A fatal neurological and reproductive system disease was recently discovered among three siblings. The main features are visceral autonomic dysfunction, severe postnatal neurological abnormalities, visual impairment, male testicular dysplasia, and sudden death due to brainstem-mediated cardiopulmonary arrest in infancy. Whole-exome sequencing revealed that homozygous frameshift variants in TSPYL1 (TSPY Like1) lacking the nucleosome assembly protein domain had abnormal transport in cells, resulting in prolonged S and G2 phases of cells and a reduced cell proliferation rate. Knockout of TSPYL1 in zebrafish can mimic the related phenotypes of early mortality, neurogenesis defects, and cardiac dilatation in patients (Buyse et al., 2020). This case study suggests a related idea for functional research on TSPY.

In addition, TSPY is considered a proto-oncogene that can antagonize its X-linked homologue (TSPX) (Li et al., 2017). TSPY can also cause a significantly higher risk of gonadal germ cell carcinoma in people with sexual development disorders than others. The main reason is that gonadalomas in the GBY region of the Y chromosome involve germ cells that are at risk when the embryonic development stage is blocked and affect the degree of testisation and gonadal maturity (Looijenga et al., 2019). The current study suggests that TSPY is the only GBY candidate gene on the short Y arm (Vogt et al., 2019). Studies have shown that TSPY is highly expressed in most of the testicular germ cell tumours evaluated and various somatic cancers, including hepatocellular carcinoma, melanoma and prostate cancer, leading to significantly higher morbidity and mortality in men than in women (Kido and Lau, 2019). The introduction of the human Y chromosome can inhibit the tumorigenicity of the prostate cancer cell line PC-3. A high-density bacterial artificial chromosome (BAC) microarray test containing 178 BAC clones from the human Y chromosome showed that the most prominent observation in prostate cancer specimens is the deletion of Yp11.2, which contains the TSPY tandem gene array (Vijayakumar et al., 2006).

### 2.3 Other Regions

In addition to these classic CNVs in the repeated regions, the Y chromosome also has a variety of CNVs, such as the deletion of AMELY in the Yp11.2 region. As a marker for sex determination in the identification of forensic evidence, the deletion of the AMELY region was quickly discovered and reported. Not only was there a large number of cases, but the types of deletions were also very diverse. In 2007, Jobling et al. detected and classified the related missing types in this area (Jobling et al., 2007), a large number of studies continued to be carried out, and new missing types were continuously reported (Cheng et al., 2019). However, many of the start and stop sites of the deletion map are mostly in the X-degenerate or X-transposed region and are highly homologous to the X chromosome or in the repetitive sequence of the Y chromosome; therefore, STS site detection cannot be performed to determine the site of the deletion. Because of the large length of the deletion region, NGS technology with a shorter read length is useless in this type of sequence detection. Most of the deletion or duplication of CNV in this region is only in Yp11.2, which does not contain the TSPY multicopy region and other ampliconic regions; therefore, it does not show reproductive disorders (Pang et al., 2021a). Except for the TSPY array, it seems that the direct relationship between CNV in the Yp region and spermatogenesis is not that obvious (Murphy et al., 2007; Nardello et al., 2021).

DYZ1 is a 3.4-kb repeat located on the long arm of the Y chromosome and ranges from 2000 to 4000 copies in normal males. DYZ1 copy number changes are directly correlated with recurrent spontaneous abortion (RSA)/early embryo growth arrest RSA/early embryo growth arrest (Yan et al., 2011). DYZ1 is highly polymorphic and has sequence differences in semen and blood samples, with good potential for use in forensic cases (Hughes et al., 2010; Ahmad et al., 2020).

Variable Charge Y (VCY) is located inside the P8 palindrome, and its protein family may be involved in the regulation of ribosome assembly during spermatogenesis (Hughes and Rozen, 2012). And due to the complex variation of palindromic sequences in the VCY region, healthy men can have 1-4 copies of VCY (Bennett-Baker and Mueller, 2017).

In addition, recombination between homologous sequences of the Y chromosome and X and/or autosomal sequences will cause the X-transposed region of the Y chromosome (which may or may not carry the sex-determining region Y gene (SRY) gene) to translocate to another chromosome. The most common is X-Y translocation, most of which is caused by abnormal crossover during paternal meiosis (Kim et al., 2017; Sha et al., 2020). Due to the small size of the Y chromosome, the remaining Y after Y chromosomal recombination is very easy to lose and causes Y chromosome deletion. The translocation of the Y chromosome containing SRY fragments to the X chromosome will result in 45. X individuals were previously known as 45, which is X maleness with testicular disorder of sex development. SRY-negative 46, which is XY gonadal dysgenesis (previously known as 46, XY femaleness), occasionally occurs due to the loss of the short arm where SRY is located, most of which are caused by abnormal translocations between the homologous genes PRKX and PRKY (Nakashima et al., 2013). Another type of gender development disorder is 46, XX-DSD; most cases are SRY positive (SRY^+^) (90%) (McElreavey et al., 1993; Vorona et al., 2007), which usually manifests as fully differentiated male external and internal genitalia with small testes, although it occasionally manifests as SRY negative and results in unclear genitalia. The most likely pathogenesis is autosomal gene mutation/overexpression, such as overexpression caused by duplication of the SRY-Box 9 (SOX9) gene or SRY-Box 3 (SOX3) and SRY-Box 10 (SOX10) gene repeats (Sutton et al., 2011). Because SOX3 and SOX10 are highly homologous to SOX9, increased expression can mimic the function of the SOX9 gene, leading to testicular development. In addition, there are some rare cases, such as sex development disorder caused by a large number of duplications of the FGF9 gene (Chiang et al., 2013). Alternatively, loss-of-function mutations in the RSPO1 and WNT4 genes are also associated with 46, XX SRY^−^ DSD cases (Baxter and Vilain, 2013). In an autosomal-Y (A-Y) translocation, there are many different phenotypes due to the difference between Y and the involved autosomal deletion sites, which generally cause oligospermia or infertility. A Chinese male individual had a Y chromosome and 3 chromosome translocations causing infertility (no AZF region deletion). The karyotype of the patient was finally described as 46, X, der (3) t (Y; 3) (q11.22; p26). His father showed the same karyotypes as the patient. In a balanced state without significant DNA loss, the A-Y translocation may not damage male fertility and spread across generations (Signore et al., 2019; Deng et al., 2020; Jiang et al., 2020).

## 3 Discussion

At present, a large number of studies have reported the correlation between the deletion of a large segment of the human Y chromosome and spermatogenesis or cancer, but there are still many problems to be solved. Many test results show that Yq microdeletion is only detected in men with abnormal sperm diagrams but not in fertile men (Sen et al., 2013; Krausz et al., 2014), indicating that these deletions are the cause of sperm failure and lead to infertility. Studies have also found that the rate of Yq microdeletion is very high in patients with azoospermia or severe oligospermia, and the incidence of Yq microdeletion is usually lower in infertile patients with sperm concentrations higher than 5 × 10^6^/ml (Mateu et al., 2010). This finding shows that it may indeed be the regulator of sperm concentration rather than the direct cause of male infertility. These research differences show that there are differences in specific populations (Sen et al., 2013). At the same time, there are many cases of evidence confirming that certain deletions unexpectedly do not affect the fertility of the individual. Case reports have also shown that the father of an infertile male carries the same Yq deletion that causes infertility (Chang et al., 1999; Gatta et al., 2002). With the advancement of technology, researchers have discovered new types of defects and have conducted more detailed research and classification of existing defects. This new classification may promote the research progress of Y chromosome deletion and related diseases. For example, in the AZFb area, it is currently believed that the lack of this area will cause azoospermia and cause male infertility. However, some patients suspect that complete AZFb loss is suspected based on routine first analysis but still have residual sperm. Stouffs and his colleagues commented on these patients. Extension analysis of the deletion of the AZFb region can amplify the distal STS (sY1192) so that this type of deletion individuals can retain some copies of multiple copies of genes, such as PRY, RBBMY, BPY2, DAZ and some transcription units, thus allowing for residual spermatogenesis and full maturity (Stouffs et al., 2017; (Cioppi et al., 2021). To date, strong evidence has not been obtained showing an association between Yp CNV and male infertility (Krausz and Casamonti, 2017; Subrini and Turner, 2021). Although some authors hypothesized that it may act as a pro-proliferation factor in a dose-dependent manner (Krausz et al., 2010), others are sceptical (Yang et al., 2018). There may also be population stratification bias in this function (Signore et al., 2019). The different phenotypes of deletion type may be caused by the unclear scope of the deletion or the regulation of sequences in other regions or even other chromosomes (Colaco and Modi, 2018; Reddy et al., 2021; Thakur et al., 2021). Without large-scale gene sequencing or exome sequencing, it is not rigorous to rashly associate Y chromosome sequence deletion (or CVN) with spermatogenesis, male infertility, or even cancer. Especially at the molecular biology level, there are very few studies on how these genes or sequences are involved in spermatogenesis or the maintenance of sperm motility.

Satellite DNA has a high mutation rate and high polymorphism, and high individual identification ability can be achieved through multiple site combinations. It is suitable for forensic individual identification and kinship identification. At the same time, it also has great advantages in forensic genealogy analysis, especially STR elements that help reconstruct the phylogenetic history of the Y chromosome. In particular, microsatellite DNA is defined as a genetic hotspot of sequence variation, and its mutation rate is 10–100,000 times higher than that of the rest of the genome. The STR marker can form a large single copy unit with the flanking region because of its short repetitive sequence. The DNA length polymorphism generated by the change in the number of repetitions can be easily detected by PCR amplification combined with capillary electrophoresis technology. Therefore, the application of STR is extremely wide in the field of forensic medicine.

Compared with the conventional genetic markers that conform to the Mendelian law of inheritance, Y-STR used as a paternal inheritance genetic marker and mitochondrial DNA used as a maternal inheritance genetic marker are of great significance in forensic genealogy research and personal identification, especially when mitochondrial DNA has a small genome and less genetic information. Y-STR has special application value for the identification of some complex genetic relationships among ancestors, grandchildren, uncles and nephews. The AZFc region with more CNV is often involved in chromosomal rearrangements that may have major phenotypic effects, such as SF or other pathologies related to male infertility. It is of great value in forensic genetics and commonly used in forensic analysis. Some Y-STRs are located in the amplicon or interamplicon sequence of AZFc. The Y-STR deletion frequency in the amplicon or inter-amplicon sequence of AZFc is much greater than other Y-STR (Nan et al., 2021). For example, there is a case showing that the Y-STR typing result is that DYS448 has an invalid allele, and DYF387S1 has a four-allele pattern. NGS read depth analysis data confirmed that it was caused by the deletion of amplicons b1-b3 (where DYS448 is located) arising concurrently or after duplication of amplicons b2-b4 (where DYF387S1 is located). At the same time, the reduction of the copy number of the RBMY gene family and the duplication of other AZFc genes caused by the large genome rearrangement were also detected. The combined detection results of the other 16 Y-STRs found that this type of duplication/deletion event was not affected by negative selection and could be inherited for multiple generations (Ravasini et al., 2021).

Many studies have shown that the sensitivity of Yq, especially the AZF region with large repeats and palindrome sequences, to microdeletions may be related to ethnicity. Y haplogroups are mainly distinguished by Y-STR and Y-SNP. In the past, STR and STS-PCR were often used in sequence deletion studies. However, due to the limitations of genetic markers and technology itself, the inconsistent use of markers between laboratories led to data comparison, and system classification is developing slowly. In recent years, the development of technologies such as MLPA and resequencing and the joint application of multiple technologies have discovered more deletion types (Mitrofanov et al., 2021). Compared with STS-PCR, which is only suitable for the detection of deletions of single-copy sequences, other new technologies such as MALP, aCGH, resequencing and ddPCR can effectively identify many CNVs including duplication and complex CNVs that cannot be detected by STS-PCR (Liu X. -Y. et al., 2017 ; Carpinello et al., 2021). At same time, they also have higher technical and economic costs, STS-PCR is still the main method of clinical screening. Refining the classification also makes the data types increasingly complex. Therefore, although some scholars have proposed the influence of the Y chromosome genetic background (such as haplogroup) on the susceptibility to partial deletion of AZFc (Colaco and Modi, 2018; Iijima et al., 2020), there are not enough systematic data to support it.

Repetitive DNA sequences play a key role in driving karyotype evolution and genome structure (Lopes et al., 2021). At the same time, they are also involved in various basic genome functions and are closely related to a variety of diseases. They have a very wide range of applications in the field of forensic medicine and clinical testing. However, until today, the major limitations of technologies have remained a restrictive factor hindering the progress of this important biological and medical field, thus leading to the delay in making breakthroughs in the sequence analysis of the part of the genome and research in related diseases. Sequencing technology and bioinformatics pipelines that can assemble chromosomes from telomeres to telomeres are currently being adjusted and developed, as well as key methods that follow the concept of genomics to promote new ideas for the study of repetitive DNA sequences and their transcripts. The entire scientific community needs to make great efforts to evaluate the widely observed and neglected elements in these genomes and provide a sufficient theoretical basis for perfecting the in-depth study of genes and revealing the mysteries of life.

Compared with other chromosomes, the number of genes in Y chromosome is relatively small, and most of them are pseudogenes. Current studies have found that most of the genes required for spermatogenesis are not Y-linked, and also found that the deletion of the Y chromosome is directly related to different degrees of male infertility. However, the molecular mechanism of many genes, especially Y-linked genes, causing male infertility/spermia disorders is not clear. Although a large number of cases have shown that these genes are directly related to male infertility, their physiological functions have not been verified by single-gene knockout mouse models, let alone the study of the molecular mechanisms of regulation. A growing body of Y chromosome deletion data suggests that copy number changes in a gene do not explain the phenotype associated with sperm status, and there may be synergistic regulation or interaction between the effects of different genes. At present, some researchers focus on the interaction between Y chromosome genes and autosomal genes when studying the molecular mechanism of Y chromosome genes regulating life processes. Studies have shown that Y chromosome genes can participate in the regulation of the expression and function (such as the maintenance of Golgi structure) of autosomal proteins (such as GOLGA2) in the form of piRNA, and then participate in the regulation of spermatogenesis. Two mutant mouse models with partial deletion of the long arm of the Y chromosome, B10. BR-Ydel and YRIIIqdel, showed changes in GOLGA2 expression, abnormal Golgi structure and spermatogenesis disorders. At the same time, knockout of GOLGA2 in mice specifically causes male gametogenesis impairment and leads to infertility. Coincidentally, GOLGA2LY, the pseudogene of GOLGA2, exists in the AZFc region. The gene is reported to be transcribed only in the testis, but whether the protein is expressed is currently debated. Studies have found that copy number changes and gene dose effects of GOLGA2LY seem to have a certain synergistic regulation of fertility in males with the deletions of the AZFc, but more direct evidence is lacking. We speculate that genes on the long arm of the Y chromosome may act as lncRNAs to form a regulatory network with the Golgi matrix protein GOLGA2, which affects the maintenance of Golgi structure and function, and participates in the regulation of acrosome formation and spermatogenesis. Although insufficient technology development is a key factor hindering the study of repetitive DNA, to better understand and explore the physiological function of Y chromosome genes, the eyes should be more open. It should not be limited to a certain gene or a certain chromosome, but should consider the role of Y chromosome genes or sequences in the regulatory network from the perspective of dynamic progress. Through technological innovation, the study of the repeat sequence itself can provide us with detailed and solid data to study its function. However, it is impossible to explore the life process just by sequencing and alignin. Extensive case data can now link genes to disease, but this level of knowledge is not enough to understand the physiological function of genes. To explore the role of genes, to study the diagnosis, prevention and treatment of diseases, it is more necessary to understand the members involved in regulation and the mechanism of regulation, so as to find the key point between genes and diseases, and then play a real guiding role in diagnosis and treatment.
